# Developmental constraint of insect audition

**DOI:** 10.1186/1742-9994-3-20

**Published:** 2006-12-12

**Authors:** Reinhard Lakes-Harlan, Johannes Strauß

**Affiliations:** 1 Justus-Liebig Universität Gießen, Institute for Animal Physiology, Integrative Sensory Physiology, Wartweg 95, D – 35392 Gießen, Germany

## Abstract

**Background:**

Insect ears contain very different numbers of sensory cells, from only one sensory cell in some moths to thousands of sensory cells, e.g. in cicadas. These differences still await functional explanation and especially the large numbers in cicadas remain puzzling. Insects of the different orders have distinct developmental sequences for the generation of auditory organs. These sensory cells might have different functions depending on the developmental stages. Here we propose that constraints arising during development are also important for the design of insect ears and might influence cell numbers of the adults.

**Presentation of the hypothesis:**

We propose that the functional requirements of the subadult stages determine the adult complement of sensory units in the auditory system of cicadas. The hypothetical larval sensory organ should function as a vibration receiver, representing a functional caenogenesis.

**Testing the hypothesis:**

Experiments at different levels have to be designed to test the hypothesis. Firstly, the neuroanatomy of the larval sense organ should be analyzed to detail. Secondly, the function should be unraveled neurophysiologically and behaviorally. Thirdly, the persistence of the sensory cells and the rebuilding of the sensory organ to the adult should be investigated.

**Implications of the hypothesis:**

Usually, the evolution of insect ears is viewed with respect to physiological and neuronal mechanisms of sound perception. This view should be extended to the development of sense organs. Functional requirements during postembryonic development may act as constraints for the evolution of adult organs, as exemplified with the auditory system of cicadas.

## Background

Insect audition represents a fascinating example of multiple evolution in several lineages. Tympanal hearing in insects evolved more than 10 times independently from each other [1-3]. These tympanal organs are located at different sites on the insect body and have distinct designs, but with common features, like a scolopidial sense organ. These sense organs belong to a family of internal mechanoreceptors found in arthropods. Phylogenetic comparison revealed that scolopidial sense organs are in the same place in hearing and non-hearing species, while hearing species having external structures elaborated for perception of airborne sound [4,5]. Thus, the scolopidial organs of non-hearing species represent the ancestral situation. A second line of evidence is that auditory sense organs are specializations of serially organized scolopidial organs [6-8].

The multiple evolution caused different morphologies of the insect ear. Interestingly, the number of sensory cells used in audition varies to an astounding degree between taxa: from only one sensory cell in some moths to more than 1000, e.g. in cicadas [9]. These differences still await functional explanation. A hypothetical cause could be frequency discrimination. In bush crickets, the sensory cells are linearly arranged with a corresponding order in frequency tuning, resembling in this respect the alignment of hair cells in the vertebrate inner ear [10,11]. However, within the central nervous system much of the information converges onto a few known interneurons (although more might still await discovery), discarding much of the frequency fractionation [12,13]. This reduction might be explained by the main functions of insect hearing which are intraspecific communication (mate finding) and predator avoidance (especially echo-locating bats). For these purposes insects can categorize stimuli for either positive or negative phonotaxis [14]. For the cicadas it has been proposed that they possess exceptional fine-frequency resolution for frequency modulated communication signals [15]. The results are based on interneuronal recordings, not directly correlated to the sensory cells. Other causes for a large complements of sensory structures might involve ecological constraints (e.g. frequency dependent attenuation), improvement of the signal to noise ratio by sampling over many independent channels [16] or sharpening of interneuron tuning with lateral inhibition. However, the causal factors of the biodiversity of ear design are unresolved [3].

The structures and functions of nervous systems in general and sensory system in particular are subject to more than one evolutionary factor which shapes them [17]. Specific sensory structures can result from (1) a (species-) specific adaptation, (2) from evolutionary history by reflecting an ancestral condition, (3) from developmental constraints or (4) from biophysical limitations of the material. Furthermore, metabolic costs are high in neuronal structures and might act as constraint that limits evolutionary change [18]. Any of these reasons can come into consideration for organismic structure and function. For the insect ear, biophysical and biochemical limitations seems to be comparable for all ears, since they are constructed from the same elements (e.g. scolopidia, cuticular tympanal membrane). Certainly species-specific adaptation and evolutionary history influences the ear design. For example moths detect echo-locating bats with only one or two sensory cells. All adaptations considered for insect ears focus on functions in the adults. Generally, developmental constraints have not yet been considered to influence the design of insect ears, because sensitive hearing is restricted to the adults. Here we propose that functional constraints during development may act as constraints for the design of insect ears.

**Figure 1:**
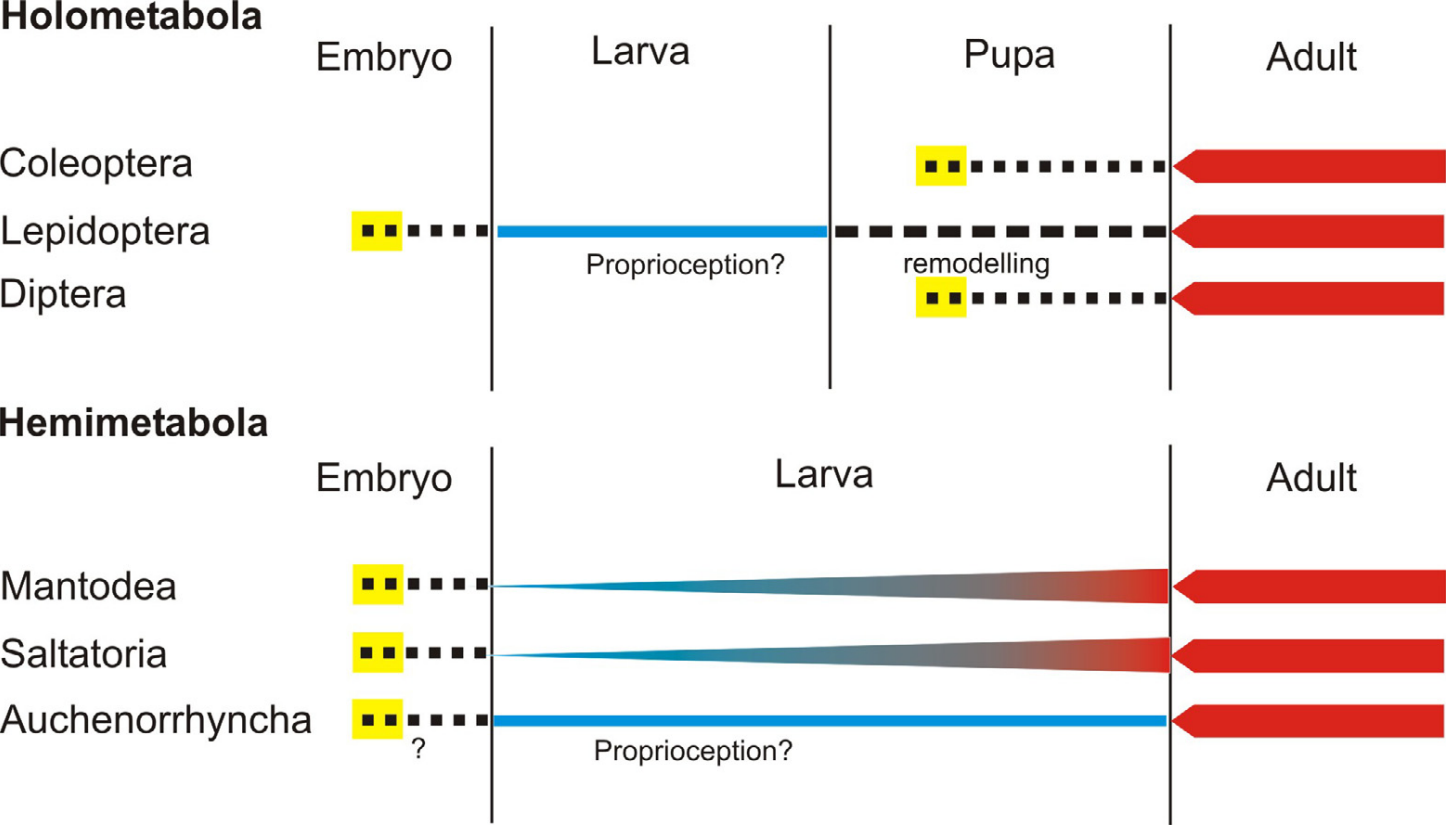
Outline of the developmental schemes for audition in six orders of insects. In the two taxa Coleoptera and Diptera of holometabolous insects the scolopidial sense organ for hearing develops during the pupal stage (generation of cells: yellow; differentiation: dotted line). Shortly after emergence the adult hearing capability is aquired (red). In at least some Lepidoptera the scolopidia develop during embryogenesis and are remodeled during the pupal stage (broken line) for the innervation of the adult tympanal organ. The larval function is probably proprioception (blue). In the Hemimetabola the scolopidia are formed during embryogenesis. In the Saltatoria and the Mantodea, the hearing capabilities develop gradually with the postembryonic larval stages (blue-red line). In the Auchenorrhyncha the cells probably develop during embryogensis and two separate functions are proposed for the larva and the adult (see text).

Insects of the different orders have also diverse ontogenetic histories. Consequently the development of the auditory organs varies largely (depictured for six orders in Figure 1). In respect to the sensitive tympanal hearing in adults, it is not surprising that in the holometabolous Diptera, the sensory cells of the ear develop during metamorphosis (Figure 1; unpublished results). The adult sensory cells have no larval precursors. The situation might be the same in Coleoptera, but it has not been studied yet. By contrast, in at least some taxa of Lepidoptera the adult organ has a predecessor organ in larval stages. This means that the scoloparia are maintained and remodelled during metamorphosis [19,20]. They develop during embryogenesis [21] and the proprioceptive larval organ measures segmental positions [20]. Thus, the tympanal organ is present only in the adult stage and the larval sensory organ serves a different function. The mechanoreceptive scolopidia can be used for different sensory modalities.

The development of the tympanal organs of the hemimetabolous Orthoptera is comparatively well investigated. All sensory cells differentiate during embryogenesis and the tympanal organs maturate gradually during postembryogenesis, [6,22-24] (Figure 1). Functionally, the sensitivity to airborne sound increases during postembryonic development [25]. This is correlated to the formation of the tympanal trachea and the thinning of the cuticle resulting in the adult tympanum [22,25]. A similar development occurs in the Mantodea [26]. In both groups of hemimetabolous insects the larvae are rather similar to the adult. However, this is different in a third group of hemimetabolous insects to be considered here, the cicadas. Larval cicadas have separated life style from the adults, resembling holometabolous insects in this respect. The different environment shaped the morphology of the animals and their sensory systems.

## Presentation the hypothesis

Here, we propose that the adult auditory system of cicadas is largely shaped by adaptations used in early developmental stages. The functional requirements of the subadult stages determine the adult complement of sensory units.

The auditory system of adult cicadas is located in the second abdominal segment and sensitive hearing is used in intraspecific communication and predator detection [27]. As mentioned above, it remains puzzling why adult cicadas have up to thousands of sensory cells. The fine-frequency resolution in the central auditory system [15] might not require such a large set of sensory neurons.

We propose adaptive purposes for the auditory system in preadult stages of the life cycle. Such a developmental influence on the adult system might be called functional caenogenesis. Per definition, a caenogenetic character is a transitory adaptive character during development [28]. The scolopidial sensory units of cicadas are probably not transitory, but their function might change and therefore the process can be called transitory.

This transitory function might explain the number of sensory cells in the sense organ. The cicada larvae have a life style of their own and occur within the soil where they suck on the tree roots. After the last moult, the morphologically different adult animals emerge from the ground. The adults live above ground on green plants performing acoustic communication for reproduction.

We propose that the scolopidial sense organ is already present during larval stages and suggest that it functions as vibration receiver. Vibration receptors and proprioceptors often have a large number of sensory units, like the femoral chordotonal organ with up to several hundred sensory cells [29]. It has been speculated that for the purpose of vibration detection cell numbers increase during evolution [30,31]. Ancestral vibration function was discussed for the numerous cells in the auditory organ in the orthopteran *Bullacris *(phylogenetic constraint) [31]. Vibration receptors and proprioceptors respond to different parameters of the mechanosensory stimuli, like acceleration, velocity, frequency and position [32] thereby requiring a large set of sensory units. Since apoptosis in scolopidial organs is unknown, the number of sensory neurons can not be downregulated from the cicada larva to the adult. The large number of neurons therefore persists after metamorphosis when the tympanal structures are differentiated, although they might not be necessary for auditory function.

## Testing the hypothesis

Experiments at different levels have to be designed to test the hypothesis, because the development of the cicada ear has yet not been investigated. Firstly, the neuroanatomy of the larval sense organ should be analyzed to detail. How many sensory cells occur in the sense organ and how is it attached to the epidermis? Another character to be analyzed is the central projection of the larval sense organ. It should be shown whether the neuronal arborizations can be found in the same areas of the central nervous systems, as the auditory receptor fibers in the adult. In other insect taxa, it was shown that the information from the serially homolog mechanoceptive organs converge in the same neuropile in the CNS and synapse onto serially homologous interneurons [7,33].

Secondly, based on the hypothetical presence of an elaborate scolopidial organ, the function of the larval sense organ should be approached neurophysiologically and behaviorally. Different vibratory and proprioceptive stimuli can be applied to the sense organ and physiological parameters, like frequency tuning, can be evaluated electrophysiologically. Behavioral experiments might elucidate the biological function. High vibration sensitivity might be important in the underground life style to avoid predation and competition. The larvae suck on tree roots and it is feasible that they compete intraspecifically for suitable sites. Aggressive or accidental interactions of the larvae might lead to fatal injuries. Thus, evasive behavior to vibratory stimuli or generation of vibratory signals should be tested.

Thirdly, the generation of the sensory cells and the rebuilding of the external structures to the adult auditory system should be investigated. This includes the questions whether new sensory cells are generated during larval stages (testable with cell cycle markers) and whether apoptosis occurs in the sense organ. Neither has been reported so far for insect auditory systems. These data might indicate a persistence of sensory neurons from the embryo to the adult.

## Implications of the hypothesis

The hypothesis presented here postulates the functional need of a vibration receptor with a high number of sensory units in larval stages of cicadas, which are retained into the adult and become transformed into an auditory system sensitive to airborne sound. Apart from the proposed hypothesis for one taxon, it has implications for the understanding of insect auditory system formation.

Usually, insect ears are studied with respect to the underlying physiological and neuronal mechanisms for sound perception. The linear gain of function during postembryogenesis may not be appropriate for the cicada. Thus, we direct the perspective away from the adult system and look at ontogenetic factors which can influence the design of an insect ear. This is a refocus of research questions, although the life cycles of many insects are not as complex as the cicada's and caenogenesis in auditory structures might not be the rule. Nevertheless, this conceptual approach re-evaluates the different life cycle stages and their importance for the adult system, replacing the adulto-centric view of animal structure with a more integral one [34].

Generally, vibrational communication is common in Hemiptera, in both larvae and adults [35,36]. Vibrational communication might represent the ancestral situation. We widen this view by the addition that larval vibration perception might shape the sensory structures of the adults. The vibratory system of treehoppers and cicadas is largely unknown [37] and the hypothesis might trigger future studies in these groups. The treehoppers and true bugs will also serve as outgroups for the studies on the functional caenogenesis proposed for cicadas. Comparative studies are also important for the understanding of the low number of sensory neurons in the subgenual organ of various insect species [38]. Certainly, vibratory behaviors can be accomplished with a few sensory cells (and perhaps an even larger complement of interneurons) [39]. The adaptive value of a low number of cells, e.g. in terms of energy costs [18] or the evolutionary constraints have to be clarified in the future.

Additionally, questions to the development of sensory systems remain open. Why can the cell numbers in many systems, like visual system or the tactile hairs, be regulated with growth of the larvae, whereas the internal sense organs can not? How can it be adaptive that large cell numbers are generated in the embryo, when the function of the sensory organ is needed only much later (years in the case of cicadas)? Neuronal cells have high metabolic costs [40,41] and therefore the maintenance of unnecessary sensory cells should be selected against. Thus, it might be feasible that sensory cells develop when they are needed – in the case of cicadas, this means they are needed already in the larvae. On the other hand, the embryonic development could be necessary when restrictions in the genetic mechanisms require that sensory mother cells occur only in the embryo, but not during postembryonic stages.

In general, this perspective forces one to carefully discriminate between primary and secondary adaptations in a studied system. It draws attention to the peculiar features of animals life styles and requires the comparative evaluation of evolutionary forces and ecological adaptations [42]. Functional requirements during development may act as constraints for the evolution of adult organs, as exemplified with the auditory system of cicadas.

## Competing interests

The author(s) declare that they have no competing interests.

## Authors' contributions

Both authors made substantial intellectual contributions to the hypothesis. RLH designed the figure. Both authors helped to draft the manuscript and approved the final manuscript.
